# A Quenched Disorder in the Quantum‐Critical Superconductor CeCoIn_5_

**DOI:** 10.1002/advs.202304837

**Published:** 2023-11-20

**Authors:** Soon‐Gil Jung, Harim Jang, Jihyun Kim, Jin‐Hong Park, Sangyun Lee, Soonbeom Seo, Eric D. Bauer, Tuson Park

**Affiliations:** ^1^ Department of Physics Education Sunchon National University Suncheon 57922 South Korea; ^2^ Department of Physics Sungkyunkwan University Suwon 16419 South Korea; ^3^ Los Alamos National Laboratory Alamos NM 87545 USA; ^4^ Department of Physics Changwon National University Changwon 51140 South Korea; ^5^ Center for Quantum Materials and Superconductivity (CQMS) Department of Physics Sungkyunkwan University Suwon 16419 South Korea

**Keywords:** critical current, peak effect, pressure, quantum‐critical superconductor, quenched disorder

## Abstract

Emergent inhomogeneous electronic phases in metallic quantum systems are crucial for understanding high‐*T*_c_ superconductivity and other novel quantum states. In particular, spin droplets introduced by nonmagnetic dopants in quantum‐critical superconductors (QCSs) can lead to a novel magnetic state in superconducting phases. However, the role of disorders caused by nonmagnetic dopants in quantum‐critical regimes and their precise relation with superconductivity remain unclear. Here, the systematic evolution of a strong correlation between superconductive intertwined electronic phases and antiferromagnetism in Cd‐doped CeCoIn_5_ is presented by measuring current–voltage characteristics under an external pressure. In the low‐pressure coexisting regime where antiferromagnetic (AFM) and superconducting (SC) orders coexist, the critical current (*I*_c_) is gradually suppressed by the increasing magnetic field, as in conventional type‐II superconductors. At pressures higher than the critical pressure where the AFM order disappears, *I*_c_ remarkably shows a sudden spike near the irreversible magnetic field. In addition, at high pressures far from the critical pressure point, the peak effect is not suppressed, but remains robust over the whole superconducting region. These results indicate that magnetic islands are protected around dopant sites despite being suppressed by the increasingly correlated effects under pressure, providing a new perspective on the role of quenched disorders in QCSs.

## Graphical abstract

The study provides new insights into understanding the role of quenched disorder in quantum‐critical superconductors through *I*–*V* characteristics. In quantum‐critical systems, magnetic droplets induced by non‐magnetic dopants are protected around the dopant site despite being suppressed by the increasingly correlated effects under pressure. Magnetic droplets nucleate around non‐magnetic dopants can act as quenched disorders that can destroy quantum‐phase transitions.

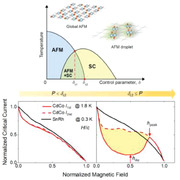

## 1 Introduction

Superconductivity and magnetism are often considered incompatible because local moments can break paired electrons (Cooper pairs).^[^ ^1^, ^2^ ^]^ However, unconventional superconducting (SC) states have been widely observed together with an antiferromagnetic (AFM) phase^[^ ^3^, ^4^, ^5^, ^6^, ^7^, ^8^, ^9^, ^10^, ^11^, ^12^, ^13^ ^]^ in strongly correlated electron systems, questioning the antagonistic relationship between them. In doped high‐*T*_c_ copper‐oxide superconductors and heavy‐fermion superconductors Ce*M*In_5_ (*M* = Co and Ir),^[^ ^4^, ^5^, ^6^, ^7^, ^8^, ^9^, ^14^, ^15^, ^16^, ^17^ ^]^ where antiferromagnetism is observed, nonmagnetic doping could lead to inhomogeneous electronic phases.^[^ ^3^, ^4^, ^18^, ^19^, ^20^, ^21^, ^22^, ^23^ ^]^ The fact that these strongly correlated electron systems are near magnetic instability suggests that the origin of the inhomogeneous phases may be associated with the interplay between the SC and AFM orders. However, the precise relationship between the competing phases and the effect of doping‐induced disorder on AFM quantum‐phase transitions in unconventional superconductors is still unclear.

**Figure 1:**
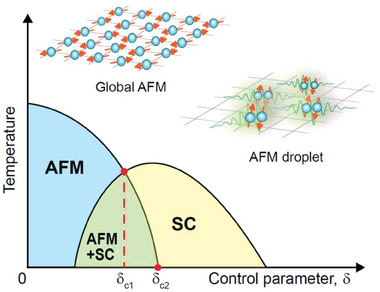
Phase diagram of quantum‐critical superconductors (QCSs) with antiferromagnetic order. High‐temperature unconventional superconductivity (SC) has been widely observed competing with antiferromagnetism (AFM). Temperature‐dependent SC and AFM are tunable via nonthermal control parameters *δ*, such as chemical doping and pressure. The Ce‐based QCS Ce*M*In_5_ (*M* = Ir, Co, and Rh) is a representative system showing the region where two phases (AFM + SC) coexist. The long‐range magnetic order abruptly disappears at the first critical point *δ*_c1_ while a superconducting dome is formed with the second critical point *δ*_c2,_ as the center, at which quantum fluctuations are thought to be crucial for mediating electron pairing.

Cadmium‐doped CeCoIn_5_ is ideal for exploring the effect of disorder on unconventional superconductors, where the SC phase coexists with the AFM state, and the spin droplets of the static magnetic orders locally formed around nonmagnetic Cd dopants overlap to induce a long‐range AFM order.^[^ ^7^, ^8^, ^9^, ^17^, ^24^, ^25^, ^26^, ^27^, ^28^, ^29^, ^30^, ^31^ ^]^ The SC transition temperature (*T*_c_) in CeCo(In_1‐_ *_x_* Cd*_x_*)_5_ decreases as the amount (*x*) of Cd increases (*T*_c_ = 2.3 K at *x* = 0). It decreases rapidly, starting from *x* ∼ 0.7%, at which the long‐range AFM order appears. For *x* > 1.5%, *T*_c_ is completely suppressed, and only the AFM transition temperature (*T*_N_) is detected.^[^ ^7^, ^16^, ^17^, ^29^, ^32^, ^33^, ^34^ ^]^ Under external pressure, as illustrated in **Figure** **1**, the global effect of Cd doping in CeCoIn_5_ exhibits a reverse behavior: *T*_N_ is suppressed, whereas *T*_c_ increases.^[^ ^7^, ^25^, ^34^ ^]^ However, nuclear magnetic resonance (NMR) indicates that applying pressure cannot reverse the Cd doping effect entirely and the inhomogeneous electronic states around the Cd dopant remain in the form of spin droplets.^[^ ^7^, ^8^ ^]^ Because probing the electronic state of matter under pressure is difficult, the effect of disorders on quantum‐critical superconductors (QCSs) such as CeCoIn_5_ remains unclear.

Here, we report the pressure‐induced evolution of the critical current (*I*_c_) and the AFM order for 1% Cd‐doped CeCoIn_5_ (a QCS, denoted as CdCo). Because pressure is a clean tuning parameter that does not introduce additional impurities,^[^ ^7^, ^12^, ^35^ ^]^ the change in the *I*_c_ under pressure should reflect how superconductivity is affected by inhomogeneous electronic states.^[^ ^36^, ^37^, ^38^, ^39^, ^40^, ^41^, ^42^, ^43^ ^]^ An anomalous behavior in the field dependence of *I*_c_ in CdCo is clearly observed near the critical pressure approximately at 12.4 kbar, at which the AFM phase is completely suppressed. Despite the increase in the applied magnetic field, a significant increase in *I*_c_ occurs near the irreversible magnetic field (i.e., the peak effect). In addition, the peak effect does not disappear despite the fact that the applied pressure is higher than the critical pressure. These results indicate that the nucleation of magnetic islands in a SC matrix serves as an impurity that is protected despite being located in a strongly correlated regime, providing new insights into the role of doping‐induced quenched disorder in QCSs.

## 2 Results and Discussion

**Figure 2:**
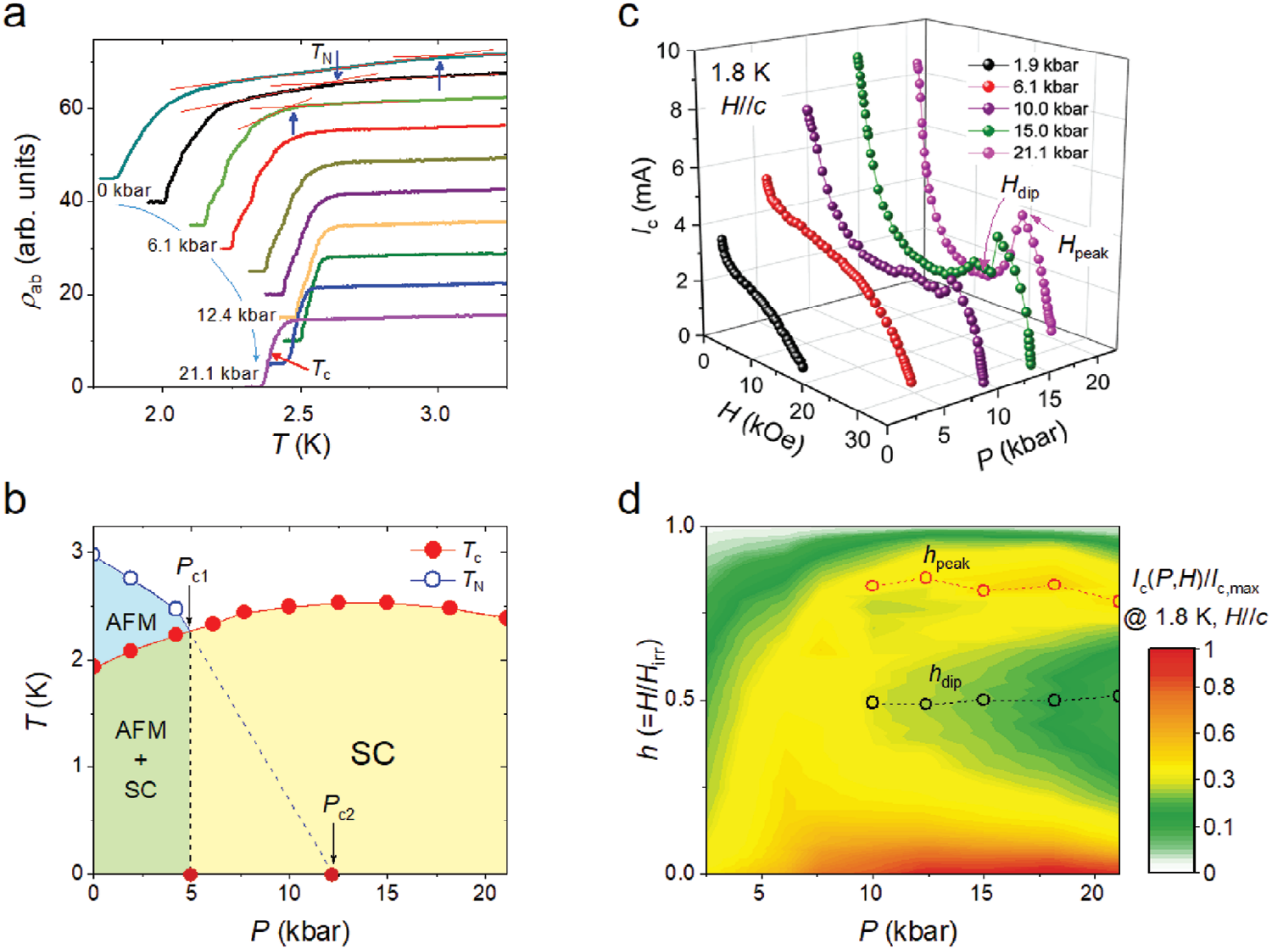
Pressure dependence of superconducting transition temperature and critical current for 1% Cd‐doped CeCoIn_5_. a) Temperature dependence of the in‐plane electrical resistivity (*ρ*_ab_) on various pressures: 0, 1.9, 4.2, 6.1, 7.7, 10.0, 12.4, 15.0, 18.2, and 21.1 kbar. Arrows indicate antiferromagnetic and superconducting phase transition temperatures, *T*_N_ and *T*_c_, respectively. *T*_c_ is determined as the middle point of the superconducting transition, and *ρ*_ab_(*T*) curves for different pressures were shifted downward for clarity. b) Pressure–temperature phase diagram of 1% Cd‐doped CeCoIn_5_ (CdCo). Here, *P*_c1_ is the critical pressure at which AFM disappears, whereas *P*_c2_ is an extrapolated AFM quantum‐critical point (QCP). c) Critical current (*I*_c_) at 1.8 K as a function of magnetic field for selective pressures. The peak effect is a sudden spike in *I*_c_ immediately before *I*_c_ = 0 at the irreversible magnetic field (*H*_irr_). The magnetic fields corresponding to the maximum *I*_c_ near *H*_irr_ and the minimum *I*_c_ before the peak effect are denoted as *H*_peak_ and *H*_dip_, respectively. d) Contour map of *I*_c_(*P*, *H*) at 1.8 K as a function of reduced magnetic field (*h*) and pressure; the magnetic field is normalized by *H*_irr_ at each pressure, and the color represents the magnitude of *I*_c_(*P*, *H*) normalized to the maximum *I*_c_ value (*I*_c,max_) among *I*_c_(*P*, *H*) measured at 1.8 K. Dashed lines are guides for the eyes.

**Figure** **2**a shows the temperature dependence of the in‐plane electrical resistivity (*ρ*_ab_) of CdCo at various pressures from ambient pressure to 21.1 kbar. Here, *ρ*_ab_(*T*) curves are rigidly shifted downwards for comparison, and *T*_N_ and *T*_c_ are indicated by arrows. With increasing external pressure, *T*_N_ ( = 3 K at ambient pressure) decreases readily, whereas *T*_c_ increases. Based on the *ρ*_ab_(*T*) curves under applied pressure, the pressure‐temperature phase diagram of CdCo for the AFM and SC states is plotted in Figure 2b. Because *T*_N_ is not observed in the *ρ*_ab_(*T*) curve at pressures above *P*_c1_, the critical pressure *P*_c2_ at which *T*_N_ goes to 0 K, is extrapolated inside the SC dome.^[^ ^7^, ^8^ ^]^ For pressures exceeding *P*_c1_, nuclear quadrupolar resonance (NQR) experiments have indicated a formation of spin droplets in CdCo compounds.^[^ ^7^, ^9^ ^]^ Studies have reported similar results for the nucleation of magnetic droplets caused by non‐magnetic impurities in Zn‐doped high‐*T*_c_ copper‐oxide superconductors and Cd‐ and Hg‐doped Ce*M*In_5_ (*M* = Co and Ir);^[^ ^4^, ^5^, ^6^, ^7^, ^8^, ^9^, ^14^, ^15^, ^16^, ^17^ ^]^ however, the role of doping‐induced disorder in QCSs is yet to be clarified.

The magnetic field (*H*) dependence of *I*_c_ in CdCo under pressure, probed via the current–voltage (*I*–*V*) characteristics (Figure S1, Supporting Information), is plotted in Figure 2c, where the *I*_c_–*H* curve at 1.8 K is shown for pressures of 1.9, 6.1, 10, 15, and 21.1 kbar. By increasing the magnetic field, *I*_c_ decreases sharply in the low‐field regime but changes curvature in the high‐field regime. For *P* > *P*_c1_, *I*_c_ exhibits an anomalous increase at the irreversible field (*H*_irr_) before reaching a zero; this is known as the “peak effect” (Figure S2, Supporting Information). In type‐II superconductors, the peak effect has been widely observed and is attributed to the interaction between vortices and disorders.^[^ ^28^, ^44^, ^45^, ^46^, ^47^ ^]^ Since the external pressure does not introduce additional disorders, the pressure‐induced peak effect in CdCo may be attributed to the magnetic clusters in the SC phase, which are nucleated in the AFM long‐range ordered regions under pressure.^[^ ^7^, ^8^, ^9^, ^24^ ^]^ At pressures exceeding *P*_c2_, the peak effect becomes more pronounced, underpinning that the magnetic islands around Cd dopants remain robust even in the pure SC phase.

The critical current *I*_c_ at 1.8 K is shown as a function of the reduced magnetic field (*h*) and pressure in Figure 2d, where *h* is the reduced field normalized by *H*_irr_ at each pressure. Here, the false color describes *I*_c_(*P*, *H*)/*I*_c,max_, where *I*_c,max_ is the zero‐field value at 18.2 kbar, which is the maximum critical current among *I*_c_(*P, H*) at 1.8 K. The characteristic magnetic fields showing a local minimum and maximum *I*_c_ near *H*_irr_ are denoted as *h*_dip_ and *h*_peak_, respectively, which are indicated by arrows in Figure 2c. The pressure dependence of *h*_dip_ and *h*_peak_ is negligible, implying that the origin of the pressure‐induced peak effect is similar at all pressures.

Spatially inhomogeneous SC phases, such as the Fulde‐Ferrell‐Larkin‐Ovchinnikov (FFLO) state, have been suggested as possible mechanisms responsible for the peak effect in strongly correlated superconductors such as UPd_2_Al_3_ and CeRu_2_.^[^ ^48^, ^49^ ^]^ In addition, the peak effect was often observed in pure CeCoIn_5_ and doped CeCoIn_5_ compounds.^[^ ^28^, ^50^, ^51^, ^52^, ^53^, ^54^ ^]^ The origin of the peak effect in pure CeCoIn_5_ was suggested to be due to the FFLO state caused by a strong Pauli paramagnetic effect.^[^ ^50^, ^52^ ^]^ On the other hand, the peak effect of Cd‐doped CeCoIn_5_ was believed to be associated with the hysteretic change in antiferromagnetic domain boundaries.^[^ ^28^ ^]^ The similarity of the peak effect of pure CeCoIn_5_ and Cd‐doped CeCoIn_5_ is that the peak effect behavior is weakened with increasing temperature,^[^ ^28^, ^50^, ^51^, ^52^ ^]^ although the origin of the peak effect is still unclear. In contrast, the peak effect behavior in this study becomes more prominent with increasing temperature and is observed over the whole superconducting phase. Therefore, our results strongly suggest that the anomalous peak effect in the QCSs, such as CdCo, may arise from separating magnetically ordered regions into magnetic islands owing to the expansion of the SC phase under external pressure.^[^ ^44^, ^45^, ^46^, ^47^, ^55^, ^56^, ^57^, ^58^ ^]^

**Figure 3:**
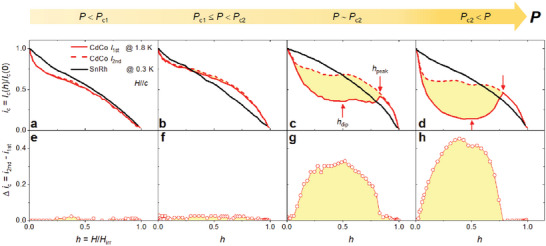
Pressure dependence on the critical current for 1% Cd‐doped CeCoIn_5_ and 4.4% Sn‐doped CeRhIn_5_. a–d) The reduced field dependence of the normalized critical current (*i*_c_) for CdCo and 4.4% Sn‐doped CeRhIn_5_ (SnRh) are plotted at representative pressures: 1.9, 6.1, 12.4, and 21.1 kbar for CdCo and 10.0, 11.4, 13.7, and 18.2 kbar for SnRh. Here, *I*_c_(*h*) is normalized to the zero‐field *I*_c_, *I*_c_(0), at each pressure. The critical pressures of *P*_c1_ and *P*_c2_ are approximately 5 kbar and 12.4 kbar for CdCo and 11 and 13.5 kbar for SnRh, respectively. The *i*_c_(*h*) of SnRh exhibits normal behavior at all pressures, whereas for CdCo, a sharp peak of *i*_c_ obviously appears in the vicinity of *H*_irr_ for the pressure near *P*_c2_, as indicated by arrows (*h*_peak_). e–h) The difference (Δ*i*_c_) between the critical currents *i*_2nd_ and *i*_1st_ of CdCo is plotted at representative pressures. Here, the *i*_1st_ and *i*_2nd_ are the *i*_c_ obtained during the first and second current sweeps at a fixed magnetic field, respectively.

**Figure** **3** shows the pressure dependence of *I*_c_(*h*) for CdCo and 4.4% Sn‐doped CeRhIn_5_ (denoted as SnRh)^[^ ^38^ ^]^ over the whole pressure range across two critical pressures *P*_c1_ and *P*_c2_. SnRh is a well‐known unconventional superconductor with a quantum‐critical point (QCP) at *P*_c2_ (≈ 13.5 kbar)^[^ ^38^, ^59^ ^]^ and has a temperature–pressure phase diagram similar to that of CdCo (Figure S3, Supporting Information). The top panel shows the normalized critical current (*i*_c_) as a function of the reduced field for CdCo and SnRh at the representative pressures of *P* < *P*_c1_, *P*_c1_ ≤ *P* < *P*_c2_, *P* ∼ *P*_c2_, and *P* > *P*_c2_ in Figure 3a–d, respectively. Here, *I*_c_(*h*) is normalized to the value of *I*_c_ at zero Tesla, i.e., *I*_c_(0). At all pressures, *i*_c_(*h*) of SnRh decreases monotonically with increasing magnetic field. However, *i*_c_(*h*) of CdCo exhibits an anomalous increase near *H*_irr_ at *P* > *P*_c1_, which becomes more pronounced at *P* > *P*_c2_, as indicated by the arrows (Figure S2 Supporting Information). The different behaviors of *i*_c_(*h*) for SnRh and CdCo may reflect the disparate pressure dependencies of the magnetic states of the two compounds. In SnRh, pressure induces an AFM QCP regardless of the Sn‐doping concentration, whereas a QCP is not observed in CdCo because the applied pressure nucleates the local spin clusters centered around the Cd dopants.^[^ ^12^, ^38^, ^59^, ^60^, ^61^ ^]^ Since slight Sn doping into CeRhIn_5_ is known to homogeneously affect the electronic structure of CeRhIn_5_, the pinning energy resulting from the small substitutional disorder due to the different atomic sizes between In and Sn atoms is believed to be insufficient to deform the entire vortex lattice of Sn‐doped CeRhIn_5_.^[^ ^38^, ^59^, ^60^ ^]^ In contrast, magnetic droplets around the Cd dopant in Cd‐doped CeCoIn_5_ can act as a quenched disorder with strong pinning energy because the size of the droplet is much larger than that of the non‐magnetic point defect induced by Sn doping into CeRhIn_5_.^[^ ^9^, ^30^, ^60^, ^61^ ^]^

When the second current sweep (*I*_2nd_) is performed, after the first sweep (*I*_1st_), at a fixed magnetic field, the peak effect revealed in *I*_1st_(*h*) of CdCo vanishes in the *I*_2nd_(*h*) curve (Figures S4,S5, Supporting Information). Figure 3e–h show the difference (Δ*i*_c_) between critical currents *i*_2nd_ and *i*_1st_ for *I*_2nd_ and *I*_1st_ at 1.8 K, respectively; a remarkably large *i*_c2_(*h*) compared to *i*_c1_(*h*) is observed for *P* > *P*_c1_ (Figure S6, Supporting Information). The large Δ*i*_c_ under pressure can be attributed to the transformation of the vortex states from unpinned (ordered) to pinned (disordered) states. The strongly pinned vortex states require relatively high critical currents to induce the motion of the vortices.^[^ ^36^, ^37^, ^44^, ^45^, ^46^, ^47^ ^]^ In the first sweep, the ordered vortex structure may be dominant owing to the elastic interaction between vortices; however, the vortices can be strongly trapped in pinning sites during flux flow when the applied current *I* is larger than the critical current *I*_1st_. In the second sweep, a critical current larger than *I*_1st_ is required to free the vortices from the pinning sites, leading to a large difference between *I*_1st_(*h*) and *I*_2nd_(*h*). *i*_1st_ and *i*_2nd_ converge at *h*_peak_, supporting the idea that the magnetic islands and the pressure‐induced flux‐pinning sites in CdCo are the sources of the peak effect.^[^ ^44^, ^45^, ^46^, ^56^, ^62^ ^]^

**Figure 4:**
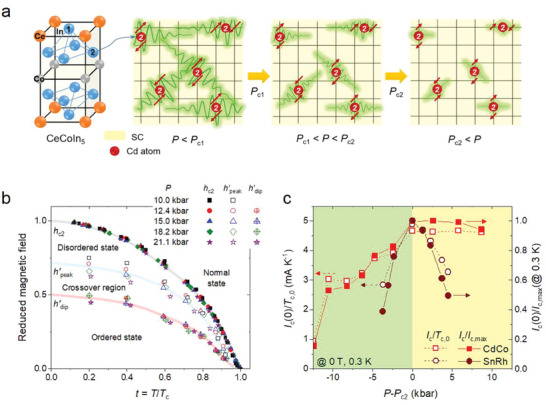
Pressure dependence of critical current and vortex phase diagrams. a) The crystal structure of CeCoIn_5_ and simple cartoons for the pressure dependence of the size of antiferromagnetic droplets in Cd‐doped CeCoIn_5_. At *P* < *P*_c1_, the long‐range antiferromagnetic order originates from the overlap of the magnetic droplets formed around the Cd dopants. The magnetic correlation length gradually decreases as the applied pressure increases, eventually nucleating the spin droplets for *P* > *P*_c1_. b) Vortex phase diagram for pressurized CdCo. Based on the *I*_1st_(*H*) curve under pressure, the vortex structure is divided into the ordered phase, crossover, and disordered phase regions. Here, the temperature dependence of *H*_c2_, *H*_peak_, and *H*_dip_ is normalized by *H*_c2_(0) at each pressure, denoted as *h*_c2_, *h″*_peak_, and *h″*_dip_, respectively. The solid lines are guides for the eyes. c) Pressure evolution of zero‐field *I*_c_(at 0.3 K) divided by *T*_c,0_ (left side) and *I*_c,max_(at 0.3 K) of CdCo (red symbols) and SnRh (brown symbols) are plotted on the left and right ordinates, respectively, where *T*_c,0_ is the critical temperature at *I*_c_ = 0 at 0 T and *I*_c,max_(at 0.3 K) is the maximum zero‐field *I*_c_ among *I*_c_(*P*) measured at 0.3 K.

The crystal structure of CeCoIn_5_ and the pressure dependence of the size of the AFM droplets in Cd‐doped CeCoIn_5_ are schematically depicted in **Figure** **4**a. The In positions substituted with Cd atoms (hole doping in CeCoIn_5_) locally induce a static AFM order on their neighboring Ce moments, because *c‐f* hybridization is locally suppressed near Cd dopants.^[^ ^7^, ^9^, ^17^, ^24^, ^25^, ^30^, ^31^, ^61^ ^]^ Because the Cd atom (5*s* ^2^) has one less *p* electron than the In atom (5*s* ^2^5*p*), Cd doping suppresses the Kondo screening of Ce moments and enhances the indirect Ruderman‐Kittel‐Kasuya‐Yosida (RKKY) exchange interaction between localized Ce moments.^[^ ^22^, ^27^, ^29^, ^30^, ^31^ ^]^ Therefore, the long‐range AFM order in Cd‐doped CeCo(In_1‐_ *_x_* Cd*_x_*)_5_ for *x* > 0.7% results in a magnetic percolation transition when the number of local magnetic droplets is greater than the critical percolation concentration.^[^ ^7^, ^8^, ^9^ ^]^ Under external pressure, however, the spin droplets are spatially isolated owing to the shrinkage in the magnetic correlation length (Figure S7, Supporting Information).

Figure 4b shows the vortex phase diagram of CdCo determined from *H*_c2_(*T*) and the peak effect in the *I*_c_(*H*) curves for pressures *P* > *P*_c1_ (Figures S6,S8, Supporting Information); here, three distinctive regions are depicted: ordered vortex, crossover, and disordered vortex. The temperature dependences of *H*_c2_, *H*_peak_, and *H*_dip_ for each vortex region are normalized by *H*_c2_(0) at each pressure, identified as *h*_c2_, *h″*_peak_, and *h″*_dip_, respectively; the lower critical‐field (*H*_c1_) region is negligible in this phase diagram because *H*_c2_ (≈ *H*_irr_) >> *H*_c1_ (*H*_c1_(0) < 1 kOe) for CeCoIn_5_.^[^ ^63^, ^64^ ^]^ Because the change in the SC coherence length (*ξ*_ab_) at pressures *P* > *P*_c1_ is insignificant, the effect of thermal fluctuations on the vortex motion associated with the peak effect hardly varies (Figure S8f, Supporting Information).^[^ ^37^, ^63^, ^65^ ^]^ In the ordered phase region, where the elastic interactions among vortices are dominant, *I*_c_ gradually decreases as the magnetic field increases (Figure S9, Supporting Information). However, the vortex–pin interaction and the plastic deformation of the vortex lattice become significant for *h* > *h″*_dip_ because the inter‐vortex distance *a*_0_ ∼ (*ϕ*_0_/*B*)^−0.5^ is inversely proportional to the magnetic field.^[^ ^36^, ^37^, ^56^, ^62^ ^]^ The effect of the pinning potential on vortices reaches its maximum around *h″*_peak_ (Figure S10, Supporting Information), resulting in a peak effect. Subsequently, *I*_c_ decreases rapidly in the disordered‐phase region. Based on the display of the peak effect over the whole SC region for *P* ≥ *P*_c2_, as shown in Figure 4b, we can conclude that the magnetic droplets nucleated around the Cd dopant in the QCS CeCoIn_5_ act as quenched disorders.^[^ ^8^, ^9^, ^66^, ^67^, ^68^, ^69^ ^]^

The pressure dependence of zero‐field *I*_c_ is plotted for CdCo and SnRh in Figure 4c, where the *x*‐axis is the relative pressure against the critical pressure *P*_c2_. *I*_c_ at 0 T, directly related to the SC volume fraction, gradually increases with pressure until reaching *P*_c2_ because of the reduction in the AFM area of CdCo (Figure S11, Supporting Information).^[^ ^38^, ^39^ ^]^ For *P* ≥ *P*_c2_, *I*_c_/*T*_c,0_ of CdCo is independent of the pressure. In contrast, for SnRh, a sharp peak in *I*_c_ is observed at *P*_c2_—as predicted for QCSs such as heavy‐fermion, iron‐based, and high‐*T*_c_ copper‐oxide superconductors—^[^ ^38^, ^39^, ^40^, ^41^, ^42^, ^43^ ^]^ because quantum fluctuations become crucial for determining the critical SC properties. Therefore, the saturation behavior of CdCo suggests that the AFM quantum‐phase transition at the putative critical point *P*_c2_ is avoided owing to the strong influence of the magnetic islands as quenched disorders.^[^ ^8^, ^66^, ^67^, ^68^ ^]^ Here, *T*_c,0_ is the critical temperature for *I*_c_ = 0 at 0 T.

## 3 Conclusion

In summary, we reported the emergence of the peak effect in the Cd‐doped CeCoIn_5_ at pressures above *P*_c1_, where the long‐range AFM state was suppressed. The lack of evidence for the AFM QCP in transport studies indicates that the antiferromagnetically ordered regions shrink spatially, therefore nucleating the localized droplets of AFM orders near the Cd dopants. With a further increase in the pressure, the peak effect is anomalously enhanced and remains robust even at *P* > *P*_c2_, indicating that the spin droplets are persistent and act as quenched disorders even in the SC background. These results underscore that Cd doping in CeCoIn_5_ leads to a nanoscale electronic‐phase separation accompanied by a significant change in local electronic states, providing insights into the role of nonmagnetic dopants and the correlation between the competing SC and AFM states in the QCSs.

## 4 Experimental Section

### Measurement Outline

High‐quality single crystals of 1% Cd‐doped CeCoIn_5_ (CdCo) were prepared via the indium (In) self‐flux method.^[^ ^7^, ^16^, ^70^ ^]^ Excess In on the surfaces of the single crystals was removed by etching in dilute hydrochloric (HCl) acid. Details of 4.4% Sn‐doped CeRhIn_5_ (SnRh) were described elsewhere.^[^ ^38^ ^]^ The pressure was applied using a hybrid clamp‐type pressure cell with Daphne oil 7575 as a pressure‐transmitting medium. The pressure was determined by monitoring the shift in *T*_c_ of high‐purity lead (Pb). The current–voltage (*I*–*V*) characteristics and electrical resistivity (*ρ*) as a function of the magnetic field under pressure for CdCo were measured using a HelioxVL system (Oxford Instruments, UK) with a 12 T superconducting magnet (IPS120‐10, Oxford Instruments, UK) and a physical property measurement system (PPMS 9 T, Quantum Design, USA). For the *I*–*V* characteristics, an electrical current was generated using an ultra‐sensitive current source (Keithley 6221, Tektronix, Inc., USA), and the voltage was measured using a nanovoltmeter (Keithley 2182A, Tektronix, Inc., USA). The electrical resistivity *ρ*(*T*, *H*) of the HelioxVL system was measured using a resistance bridge (Model 372 AC, Lake Shore Cryotronics, Inc., USA) with a current of 100 µA.

### Measurement Details

The *I*–*V* characteristic curves were measured using a pulse mode in the current source (Keithley 6221) and a nanovoltmeter (Keithley 2182A) to minimize Joule heating during the measurement. Here, a pulse‐current duration of 2 ms was used with a repetition rate of 500 ms pulse^−1^. A standard four‐probe technique was used to measure the *I*−*V* and *ρ*(*T*) curves, and a good ohmic contact with the samples was achieved using a platinum (Pt) wire and silver epoxy. The critical current (*I*_c_) was determined from the criterion of 1 µV, and the field dependence of *I*_c_ for the first and second current sweeps (upward ramps) *I*_1st_ and *I*_2st_, respectively, were obtained at a fixed magnetic field. To investigate the magnetic field dependence of *I*_c_ at various temperatures, the sample was first zero‐field cooled from a temperature well above *T*_c_ to each target temperature. Then, magnetic fields were applied perpendicular to the *ab* plane of the samples, and each target magnetic field was approached using a sweep rate of 30 Oe s^−1^. The upper critical field (*H*_c2_) was estimated from the *ρ*(*T*, *H*) curve determined from the midpoint of the SC transition. The dimensions of the measured CdCo crystals were 1055 µm × 120 µm × 25 µm.

## Conflict of Interest

The authors declare no conflict of interest.

## Supporting information

Supporting Information

## Data Availability

The data that support the findings of this study are available from the corresponding author upon reasonable request.
