# Supplementary material for: A Quenched Disorder in the Quantum‐Critical Superconductor CeCoIn5
Source: Adv Sci (Weinh). 2023 Nov 20;11(1):2304837. doi: 10.1002/advs.202304837 (PMC10767398; doi:10.1002/advs.202304837)
Supplement: Supplementary file 1 — Supporting Information [file ADVS-11-2304837-s001.pdf]

ADVANCED SCIENCE
Open Access

Supporting Information

for *Adv. Sci.*, DOI 10.1002/advs.202304837

A Quenched Disorder in the Quantum-Critical Superconductor $CeCoIn_5$

Soon-Gil Jung, Harim Jang, Jihyun Kim, Jin-Hong Park, Sangyun Lee, Soonbeom Seo, Eric D. Bauer and Tuson Park**

Supporting Information

A quenched disorder in the quantum-critical superconductor $CeCoIn_5$

Soon-Gil Jung, Harim Jang, Jihyun Kim, Jin-Hong Park, Sangyun Lee, Soonbeom Seo, Eric D. Bauer, and Tuson Park**

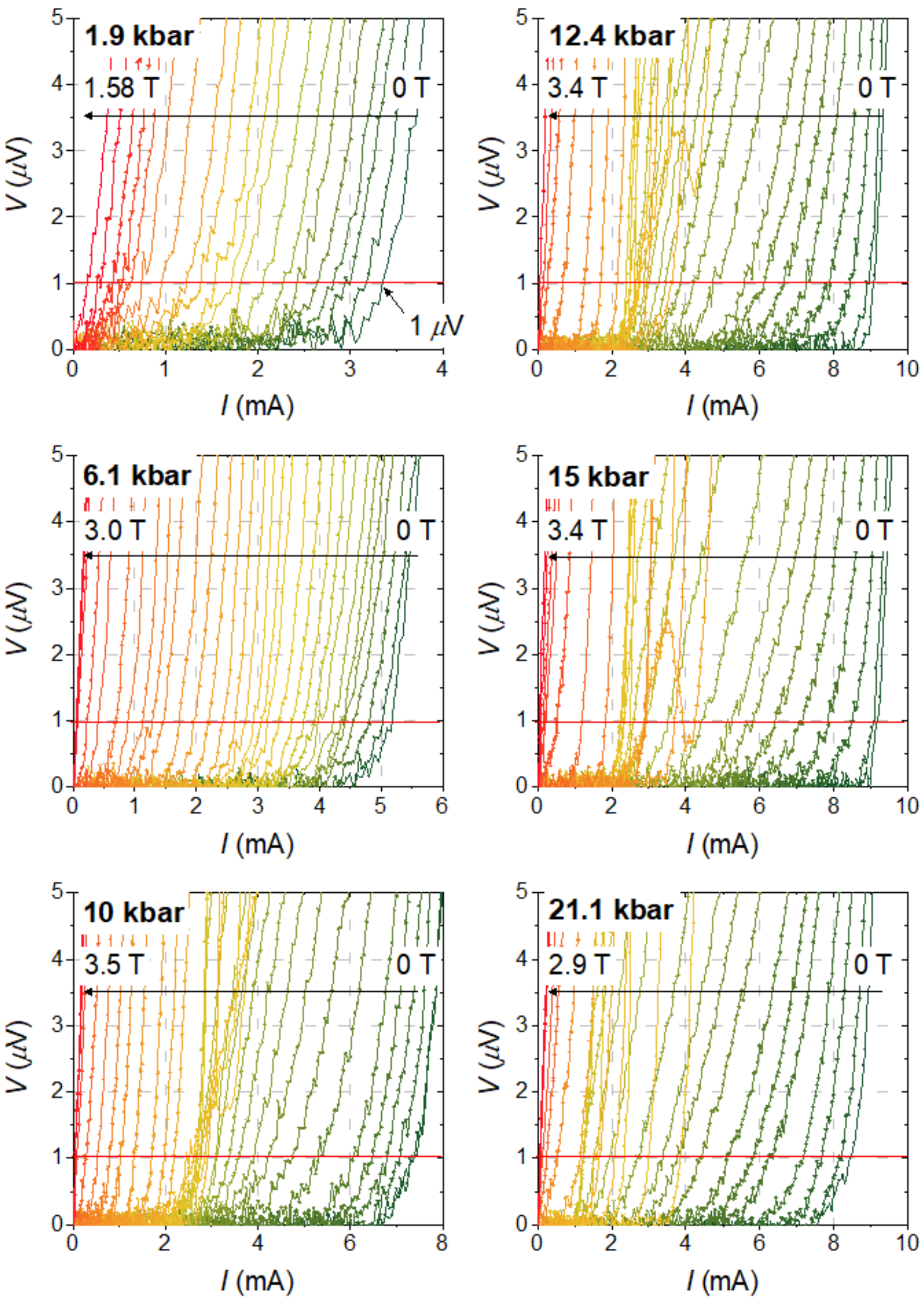

Figure S1 Current-voltage characteristics at 1.8 K for 1% Cd-doped $CeCoIn_5$ under various pressures. Magnetic field dependencies of current-voltage (*I*–*V*) characteristic curves at pressures of 1.9, 6.1, 10.0, 12.4, 15.0, and 21.1 kbar. Here, the *I*–*V* curves at 1.8 K for the first current sweep are shown as representative. The critical current (I_c) is determined using the 1 μV criterion, indicated by a horizontal red line. Black arrows represent the field variation over which the *I*–*V* curves were measured.

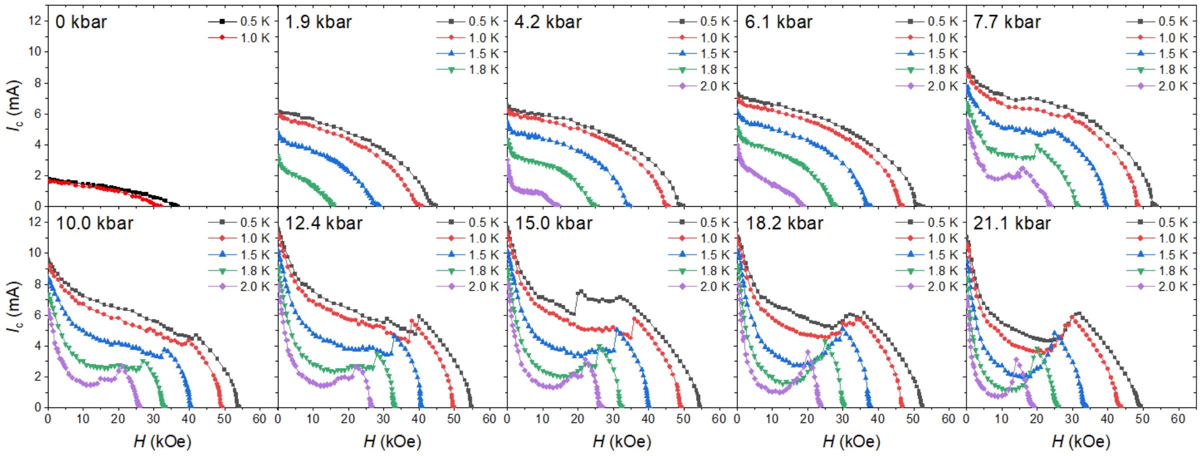

Figure S2 Peak effect in pressurized 1% Cd-doped $CeCoIn_5$. Pressure evolution of I_c with respect to the magnetic field for selective temperatures obtained from the first run of the current sweep. An anomalous increase in $I_c(H)$ appears for temperatures $T > 1.0$ K at 7.7 kbar and in all superconducting regions for $P > 10.0$ kbar, representing the emergence of the peak effect after the breakup of coupling of magnetic droplets.

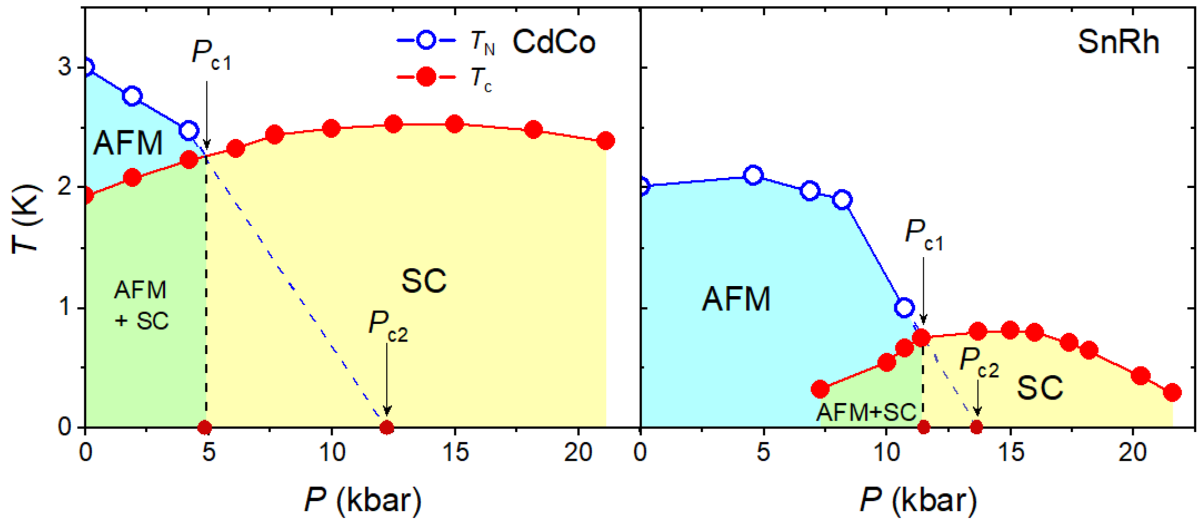

Figure S3 Comparison of phase diagrams for 1% Cd-doped $CeCoIn_5$ and 4.4% Sn-doped $CeRhIn_5$. The 1% Cd-doped $CeCoIn_5$ (CdCo) and the 4.4% Sn-doped $CeRhIn_5$ (SnRh) [S1] have similar pressure-temperature phase diagrams despite different T_N and T_c values. T_N vanishes at P_{c1} and is extrapolated to zero Kelvin at P_{c2}.

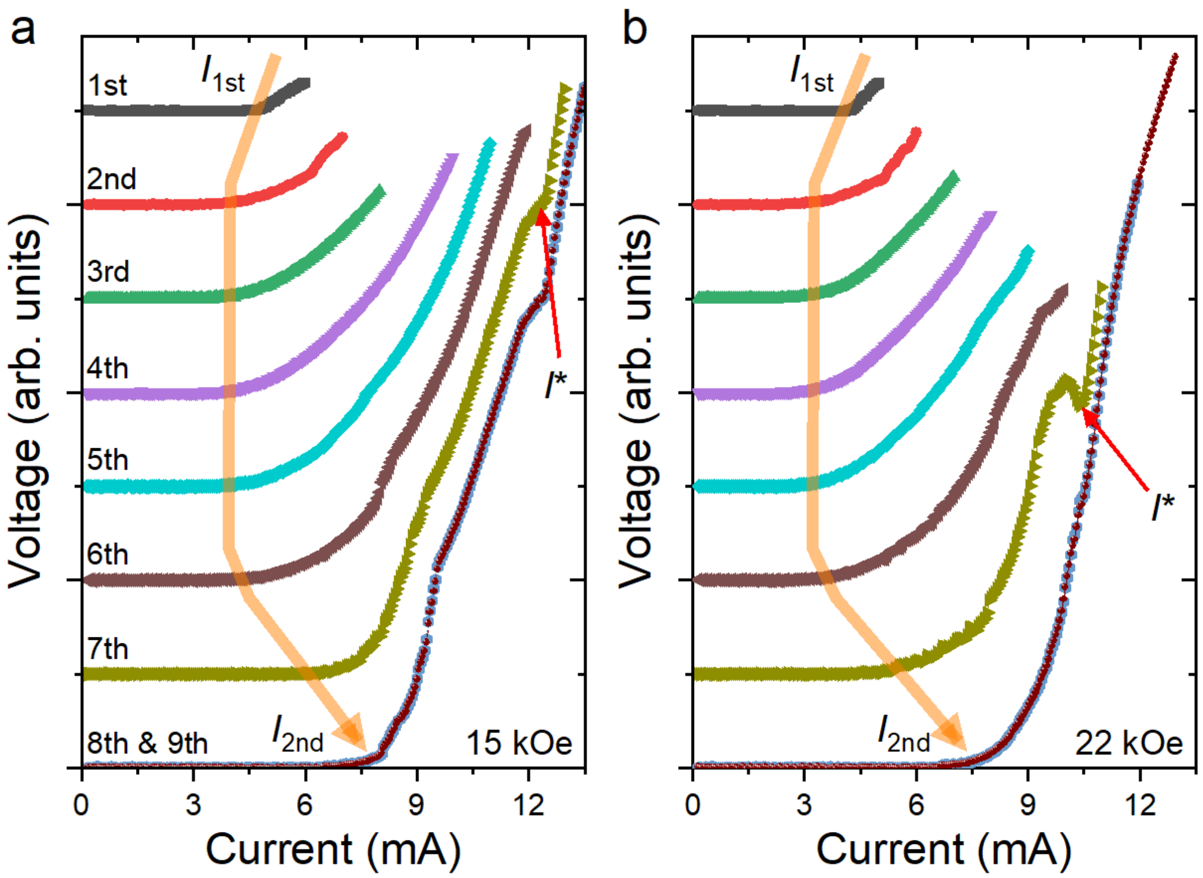

Figure S4 Current-voltage curves of sequential current sweeps at fixed magnetic fields for 1% Cd-doped $CeCoIn_5$. a) and b) *I*–*V* characteristics at a fixed magnetic field of 15 kOe and 22 kOe, respectively, at 0.5 K under the pressure of 21.1 kbar. Here, each external magnetic field was applied after zero-field cooling, and then current sweeps were performed sequentially with systematically increasing current values. The *I*–*V* curves are rigidly shifted downwards for comparison. The slight decrease in I_c in the 2nd to 6th current sweeps compared to I_{1st} is believed to be due to the unstable vortex state driven by the first current sweep (1st). However, when the applied current is greater than *I**, the voltage value exhibits an anomalous kink or dip. Since the vortices can be strongly trapped by strong pinning sites, a current higher than I_{1st}, i.e. I_{2nd}, is required to generate flux flow.

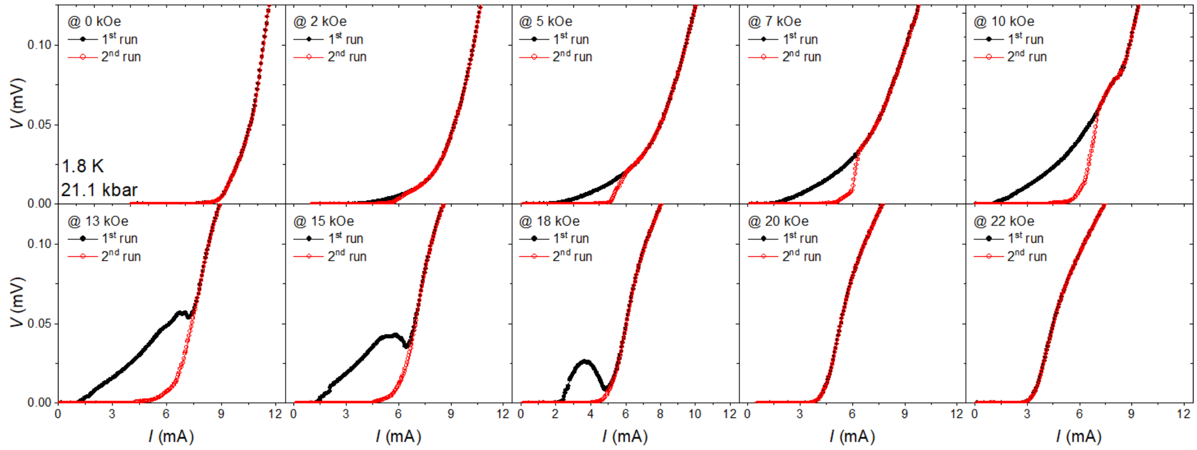

Figure S5 The first and second current sweeps for 1% Cd-doped $CeCoIn_5$ at 21.1 kbar. *I*–*V* characteristic curves for the pressurized CdCo at 1.8 K and selective magnetic fields. The difference between *I*–*V* curves measured by the first and second current sweep gradually increases with increasing applied magnetic field but begins to be suppressed at the boundary of 13 kOe (= H_{dip}). Subsequently, the first and second sweep *I*–*V* curves merge at 20 kOe, corresponding to H_{peak}. The disparate *I*–*V* curves for the first and second sweeps lead to different critical currents: i_{1st} and i_{2nd}. The third current sweep has the same *I*–*V* curve as that of the second sweep and is thus not shown here. Both the first and second sweeps were performed with increasing electrical current.

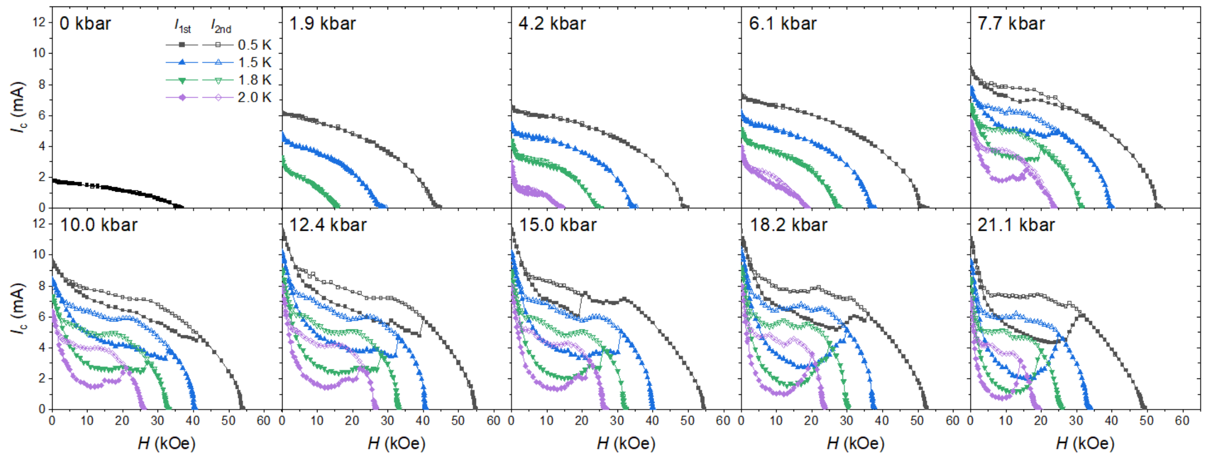

Figure S6 Magnetic field dependence of the critical current for 1% Cd-doped $CeCoIn_5$ under various pressures. The critical currents corresponding to I_{1st} and I_{2nd} with respect to the magnetic field for selective temperatures are obtained from the first and second sweeps, respectively. The difference between the I_{1st} and the I_{2nd} at the same magnetic field is associated with the change in vortex configuration from the unpinned to the pinned state, and the local magnetic droplets formed by the applied pressure are believed to be the main source of flux pinning.

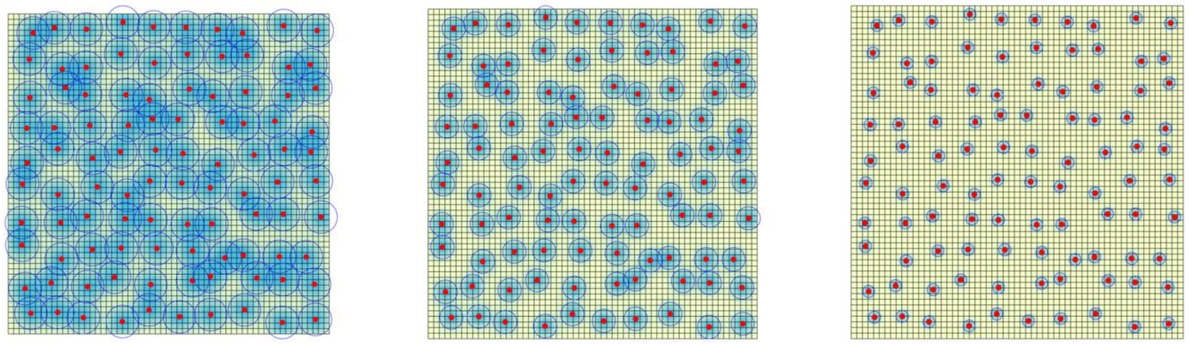

Figure S7 Simple cartoons for the pressure dependence of the droplet size in 1% Cd-doped $CeCoIn_5$. The effective droplet size (d) at ambient pressure is estimated considering the critical doping concentration (x ~ 0.7%) for the emergence of long-range AFM in $CeCo(In_{1-x}Cd_x)_5$ [S2, S3]. The unit cell of the parent $CeCoIn_5$ is denoted by a small black mesh constituting a 60 × 60 unit cell, with red symbols indicating the Cd atoms doped in the In(2) positions. In addition, for the substitution of Cd into the positions of In, we only considered In(2) sites because the In(1) sites replaced by Cd atoms are unrelated to the static magnetic order [S4, S5]. Subsequently, the average distance (a_{avg}) between Cd dopants, which is the same as with d at ambient pressure, was determined from the inverse cube root of the Cd concentration; for x = 0.7%, $a_{avg} = (7/2000)^{-1/3} \approx 6.59$ unit cell [S6]. Here, we assumed that the proportions of the Cd atom on In(1) and In(2) sites were equal. The change in the droplet size against applied pressure is calculated based on the pressure dependence of the spin-lattice relaxation rate $1/T_1$ for CdCo in Ref. S6: $d \approx$ 6.59, 6.01, 4.16, and 2.11 unit cells at 1 bar, 1.9 ($P < P_{c1}$), 6.1 ($P_{c1} < P < P_{c2}$), and 12.4 kbar ($P_{c2} \leq P$), respectively.

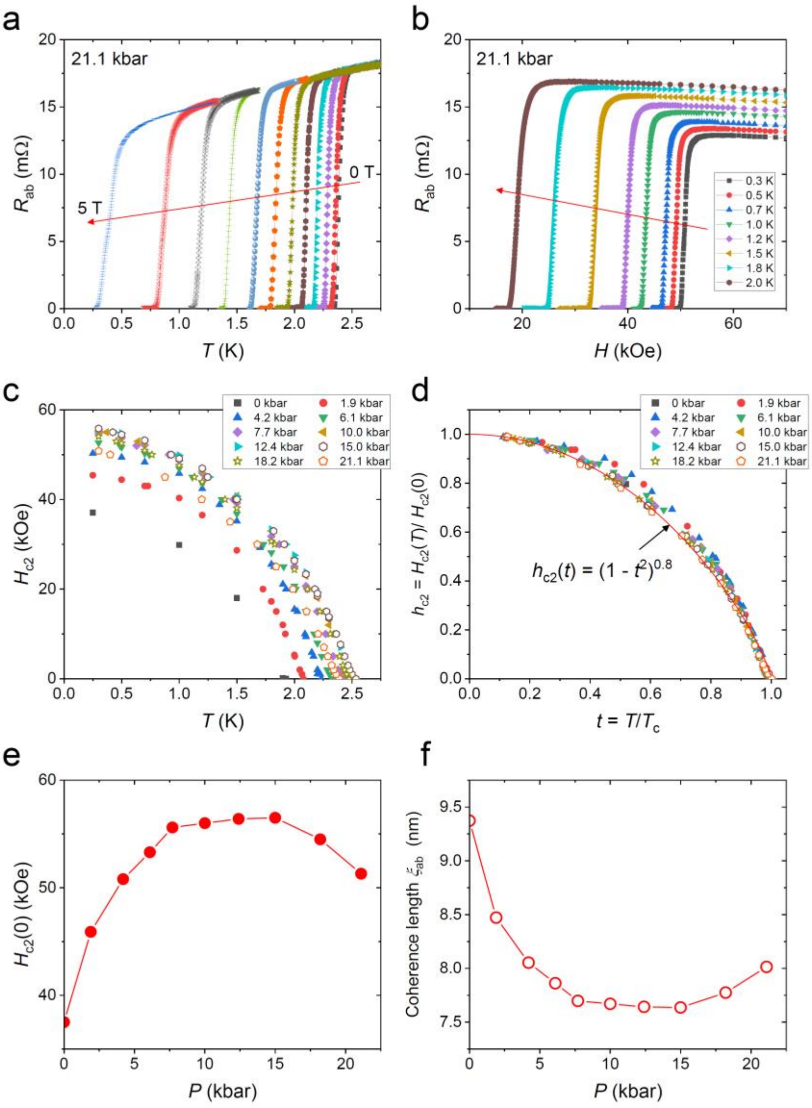

Figure S8 Pressure dependence of the upper critical field for 1% Cd-doped CeCoIn$_5$. The upper critical field (H_{c2}) for CdCo under pressure was estimated from the temperature and magnetic field dependences of in-plane resistance (R_{ab}). a) and b) show representative data measured at 21.1 kbar for the R–T curve and magnetoresistance at various magnetic fields and temperatures, respectively. $H_{c2}(T)$ is determined from the midpoint of the superconducting transition following the red arrows. c) Temperature dependences of H_{c2} and d) reduced upper critical field (h_{c2}) as a function of normalized temperature (t) for CdCo under various pressures. The H_{c2} at zero Kelvin, $H_{c2}(0)$, was determined from the empirical curve $H_{c2}(T) = H(0)[1–(T/T_c)^2]^{0.8}$, as plotted in d) with a red line. e) and f) show H_{c2} at zero Kelvin, $H_{c2}(0)$, and superconducting coherence length (ξ_{ab}) as a function of pressure, respectively. Here, ξ_{ab} is estimated from the relation $H_{c2} = \phi_0/2\pi\xi^2$.

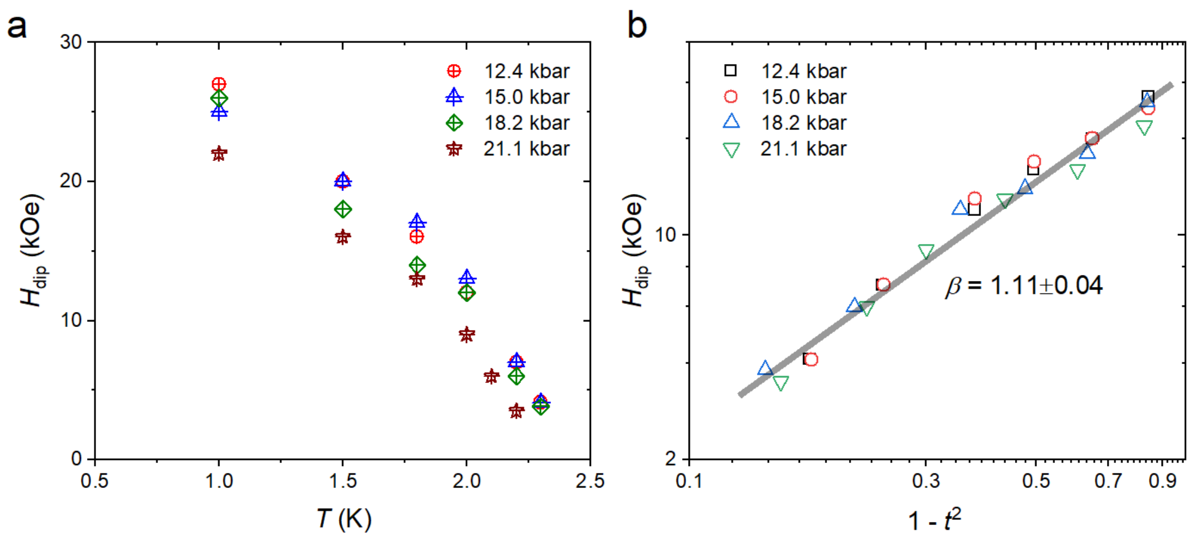

Figure S9 Temperature dependence of H_{dip} at $P \geq P_{c2}$. a) Temperature dependences of H_{dip} ($P \geq P_{c2}$), at which $I_c(H)$ has a minimum value before the peak effect. b) The H_{dip} at various pressures shows the same temperature dependencies, $H_{dip}(t) \propto (1-t^2)^{\beta}$ with $\beta = 1.11 \pm 0.04$, as denoted by the solid line. Since the energy of vortex-vortex repulsion is proportional to $1/\lambda^2$ and $CeCoIn_5$ has an in-plane penetration depth $\lambda_{ab}(t) = \lambda_{ab}(0)/(1-t^{2.17})^{-0.5}$, the H_{dip} can be thought to be associated with the elastic interaction between vortices [S7, S8].

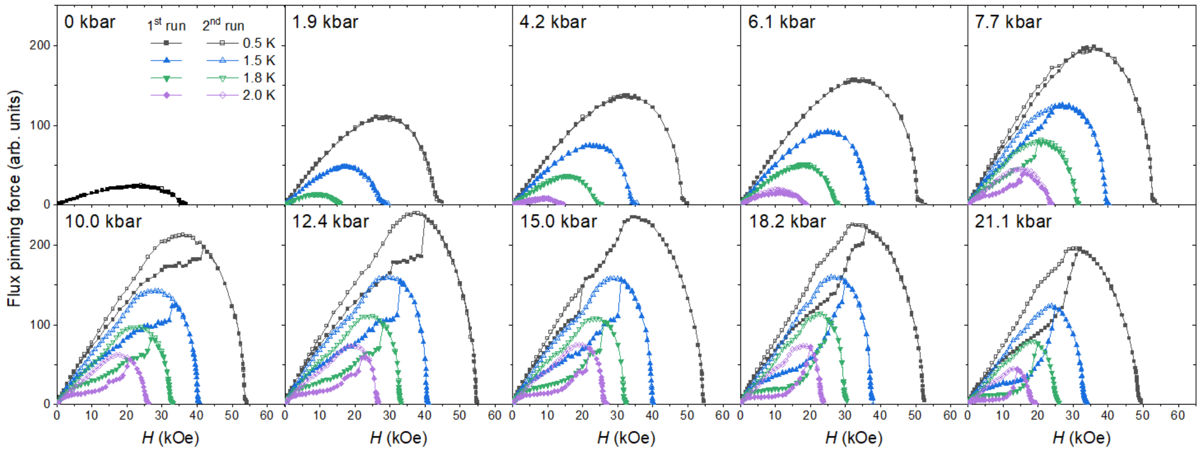

Figure S10 Pressure dependences of flux-pinning force for 1% Cd-doped $CeCoIn_5$ under various pressures. The magnetic field dependence of flux-pinning force (F_p) is obtained from the relation $F_p(H) = I_c(H) \times H$. The H_{peak}, at which $I_c(H)$ exhibits an anomalous peak in the vicinity of H_{c2} in the pressurized CdCo, is located near the F_p maximum point, indicating that the softening of the vortex lattice induces a rapid decrease in $I_c(H)$ at $H > H_{peak}$, accompanied by a disordered vortex phase [S9, S10].

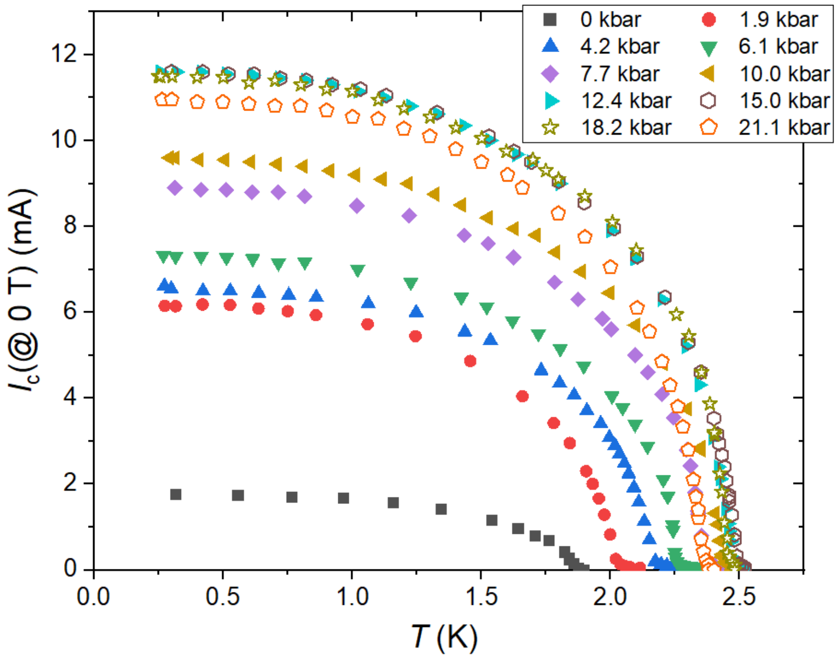

Figure S11 Temperature dependence of the critical current at zero Tesla for 1% Cd-doped $CeCoIn_5$ under various pressures. The I_c, measured at 0 T, shows a significant improvement up to the critical pressure at which long-range AFM is assumed to disappear, though the increase in T_c is insignificant. Because the supercurrent carrying ability of superconductors is related to the superfluid density [S11], suppressing the antiferromagnetic area in CdCo through the application of pressure can enhance I_c(at 0 T) owing to an increase in superconducting volume fraction.

Supplementary References

[S1] S. –G. Jung, S. Seo, S. Lee, E. D. Bauer, H. –O. Lee, T. Park, *Nat. Commun*. **2018**, 9, 434.

[S2] M. Nicklas, O. Stockert, T. Park, K. Habicht, K. Kiefer, L. D. Pham, J. D. Thompson, Z. Fisk, F. Steglich, *Phys. Rev. B* **2007**, 76, 052401.

[S3] K. Chen, F. Strigari, M. Sundermann, Z. Hu, Z. Fisk, E. D. Bauer, P. F. S. Rosa, J. L. Sarrao, J. D. Thompson, J. Herrero-Martin, E. Pellegrin, D. Betto, K. Kummer, A. Tanaka, S. Wirth, A. Severing, *Phys. Rev. B* **2018**, 97, 045134.

[S4] H. Sakai, F. Ronning, J. –X. Zhu, N. Wakeham, H. Yasuoka, Y. Tokunaga, S. Kambe, E. D. Bauer, J. D. *Phys. Rev. B* **2015**, 92, 121105(R).

[S5] K. Haule, C. –H. Yee, K. Kim, *Phys. Rev. B* **2010**, 81, 195107.

[S6] S. Seo, X. Lu, J. –X. Zhu, R. R. Urbano, N. Curro, E. D. Bauer, V. A. Sidorov, L. D. Pham, T. Park, Z. Fisk, J. D. Thompson, *Nat. Phys.* **2014**, 10, 120–125.

[S7] L. Howald, A. Maisuradze, P. D. de Rèotier, A. Yaouanc, C. Baines, G. Lapertot, K. Mony, J. –P. Brison, H. Keller, *Phys. Rev. Lett.* **2013**, 110, 017005.

[S8] J. –C. Wei, T. –J. Yang, *Jpn. J. Appl. Phys.* **1996**, 35, 5696–5700.

[S9] S. Bhattacharya, M. J. Higgins, *Phys. Rev. B* **1994**, 49, 10005.

[S10] C. J. Olson, C. Reichhardt, S. Bhattacharya, *Phys. Rev. B* **2001**, 64, 024518.

[S11] E. F. Talantsev, J. L. Tallon, *Nat. Commun*. **2015**, 6, 7820.
